# Genome-Wide Identification and Expression Analysis of the Casparian Strip Membrane Domain Protein-like Gene Family in Peanut (*Arachis hypogea* L.) Revealed Its Crucial Role in Growth and Multiple Stress Tolerance

**DOI:** 10.3390/plants13152077

**Published:** 2024-07-26

**Authors:** Yating Su, Jieyun Fang, Muhammad Zeeshan Ul Haq, Wanli Yang, Jing Yu, Dongmei Yang, Ya Liu, Yougen Wu

**Affiliations:** 1 School of Breeding and Multiplication (Sanya Institute of Breeding and Multiplication), School of Tropical Agriculture and Forestry, Hainan University, Sanya 572025, China; 2 Key Laboratory for Quality Regulation of Tropical Horticultural Crops of Hainan Province, Hainan University, Haikou 570228, China

**Keywords:** cultivated peanut, CASPs, Casparian strip, stress, bioinformatics

## Abstract

Casparian strip membrane domain proteins (CASPs), regulating the formation of Casparian strips in plants, serve crucial functions in facilitating plant growth, development, and resilience to abiotic stress. However, little research has focused on the characteristics and functions of *AhCASPs* in cultivated peanuts. In this study, the genome-wide identification and expression analysis of the *AhCASPs* gene family was performed using bioinformatics and transcriptome data. Results showed that a total of 80 *AhCASPs* members on 20 chromosomes were identified and divided into three subclusters, which mainly localized to the cell membrane. Ka/Ks analysis revealed that most of the genes underwent purifying selection. Analysis of *cis* elements suggested the possible involvement of *AhCASPs* in hormonal and stress responses, including GA, MeJA, IAA, ABA, drought, and low temperature. Moreover, 20 different miRNAs for 37 different *AhCASPs* genes were identified by the psRNATarget service. Likewise, transcriptional analysis revealed key *AhCASPs* responding to various stresses, hormonal processing, and tissue types, including 33 genes in low temperature and drought stress and 41 genes in tissue-specific expression. These results provide an important theoretical basis for the functions of *AhCASPs* in growth, development, and multiple stress resistance in cultivated peanuts.

## 1. Introduction

Cultivated peanut (*Arachis hypogaea* L.), also known as the peanut or long-lived fruit, is an annual herbaceous plant of the legume *Arachis*. Peanuts are native to South America, including Brazil, Peru, and other countries. They are mainly distributed in Asia, Africa, and America, as well as in tropical and subtropical agricultural climate zones [1]. Peanuts are an important oil crop with economic value worldwide [2]. Peanuts are renowned for their nutritional richness, containing oil (40–60%), protein (10–20%), carbohydrates, minerals, essential vitamins, antioxidants, and unsaturated fatty acids [3]. In addition, peanuts are also the main source of important medicinal compounds such as sterols, arginine, resveratrol, and flavonoids [4]. These compounds effectively prevent or treat chronic diseases, including cardiovascular disease, diabetes, and cancer, and have high medicinal value [5]. Peanuts have been introduced and cultivated in over 100 countries or regions worldwide, including China, the US, and India [6]. China is a significant producer and consumer of peanuts in the global market, with an area of about 5 million hectares under cultivation and an annual yield of more than 18 million tons [7,8]. However, peanuts often suffer from abiotic stresses (including drought, salt, extreme temperature, and heavy metal stress) during their growth and development, seriously affecting peanut germination, development, flowering, and fruiting processes. These issues contribute to reduced yield and quality of peanuts and thus limit the peanut industry’s growth in terms of sustainability and high-quality production [9]. Therefore, an in-depth study of the peanut stress response and regulation of molecular mechanisms is urgently needed to ensure high-quality cultivation and production of peanuts and create new stress-tolerant germplasm.

Abiotic stress is a general term for various environmental factors detrimental to plant growth, such as drought, salt, extreme temperature, and heavy metals [10]. When a peanut plant senses abiotic stress, it initiates a series of early stress-responsive genes that activate or repress the expression of downstream-associated genes/transcription factors via different signaling pathways [11]. These stress-responsive and regulatory genes regulate various physiological and biochemical responses in plants, such as osmotic pressure regulation, stomatal opening and closing, antioxidant system activation, etc. For drought stress, drought signaling induces the expression of transcription factors such as DREB [12], WRKY [13], NAC [14], bHLH [15], and bZIP [16], which, respectively, regulates the expression of downstream drought resistance genes, and then improves the drought resistance ability of crops. Meanwhile, it is also reported that functional genes related to water transport (PIP, TIP), E3 ligase SIZ 1, and dehydrating protein DHN are induced under drought conditions and resist drought stress by regulating the water potential, osmotic potential and ROS accumulation [17]. Currently, known anti-drought signaling pathways include the ABA signaling pathway, Ca^2+^ signaling pathway, and mitogen-activated protein kinase (MAPKs) cascade signaling pathway [18]. In resisting salt stress, the SOS pathway is crucial, with SOS1 helping plants to resist salt stress by reducing intracellular Na^+^ toxicity through the transport of Na^+^ outside the cell [19]. It has been found that transcription factors such as NAC72 [20], ERF1 [21], MYB42 [22], WRKY81 [23], etc., can respond to salt stress and activate the expression of stress-related genes, thus improving the salt-tolerant ability of crops [24]. CBFS/EREBs constitute the central component of the regulatory network that functions under low-temperature stress, and their activity is modulated by ICE1 [25]. Furthermore, LEA and HSPs proteins safeguard cells from denaturation and preserve membrane fluidity under low temperatures [26]. Previous studies have demonstrated conclusively that the formation and integrity of Casparian strips, such as *CASP1*, CIF1/2, GSO1/2, and MYB36 [27,28], play an important role in stress. What kind of genes are involved in forming and regulating peanut Casparian strip? Do these genes have stress tolerance functions? Which signaling pathways are involved? These questions need to be further answered.

The Casparian strip is a special wall structure surrounded by endothelial cells’ radial and transverse walls. It has the characteristics of bolted and lignified band thickening [29]. The main function of this structure is to screen, block, and cut off unwanted ions or macromolecules from entering the vascular column [28,30]. Current investigations have revealed that the formation and regulation of the Casparian strip encompass the intricate interplay of numerous genes and signaling pathways [31,32], such as Casparian strip domain proteins (*CASPs*) [33], leucine receptor kinase (*GSO1*/*SGN3*) [34], enhanced suberin 1 (*ESB1*) mutant [35], MYB domain protein 36 (*MYB36*) [36] and endodermis Casparian strip integrity factor (CIF1/2) [37]. As membrane proteins specific to the Casparian strip-forming region, *CASPs* feature a distinctive transmembrane topology, including the amino and carboxyl termini in the cytoplasm and conserved extracellular ring structures. These properties are critical for forming Casparian strips, ensuring their core function in the plant cell wall architecture [38]. In the *Arabidopsis* genome, 39 members belong to the *CASPs* family, while in the rice genome, 19 members have been identified [39]. In addition, there are 48 in cotton [40], 61 in banana [41], and 156 in patchouli [42]. These results indicate differences in the number of *CASPs* family members among different species. Further studies have found that *CASPs* family genes play an important role in salt tolerance [43], cold tolerance [44], and other abiotic stresses. However, the research on the biological function of plant *CASPs* genes mainly focuses on model plants, such as *Arabidopsis thaliana* and rice. The related research on peanut *AhCASPs* genes has not been reported. Regarding the peanut *AhCASPs* gene family, the number of members remains unknown. Furthermore, it is necessary to determine which specific members can respond to adversity stress. The stress regulation signaling pathways implicated in peanut *AhCASPs* also require thorough examination and investigation.

In this study, we conducted a rigorous bioinformatics analysis of the whole genome data of cultivated peanuts, resulting in the screening and identification of 80 *AhCASPs* family members. Subsequently, a comprehensive study was performed to elucidate the physicochemical properties of these proteins, the distribution across chromosomes, the promoter *cis-*acting elements, and their evolutionary expression characteristics. In addition, through the analysis of peanut transcriptome data of different treatments, plant growth regulators, and tissues, the key *AhCASPs* candidate genes involved in peanut stress (33 genes), hormone stress (40 genes), and tissue specificity (41 genes) were screened and identified. The findings of this study furnish a theoretical foundation for examining the *AhCASPs* gene family’s functionality and its significance in peanut’s response to stress conditions.

## 2. Results

### 2.1. Identification of AhCASPs Genes in Peanut

We used a two-step method to ascertain the members of the *AhCASPs* gene family in the peanut genome. The first Blast obtained 185 candidate *AhCASPs* genes based on the Pfam (PF04535) file. Subsequently, using the known *AtCASPs* protein sequence from *A. thaliana* as a query and blast alignment of the peanut proteome database, 91 candidate *AhCASPs* genes in peanuts were retrieved. The genes initially identified as potential candidates were subjected to a comparative analysis with those subsequently identified on the second occasion, and the repeated transcripts were deleted. Finally, 80 members of the *AhCASPs* gene family have been successfully identified and obtained. To understand the physical and chemical properties of 80 peanut AhCASPs proteins, we used the online analysis tool ExPASy (https://www.expasy.org/ accessed on 27 April 2024) to analyze their protein sequences comprehensively. We predicted the isoelectric point and molecular weight of peanut AhCASPs proteins (Table 1). The results indicated that the amino acid length of peanut AhCASPs protein was between 72 and 810 amino acids). The gene with the least number of amino acids was *AhCASP65* (containing 72 amino acids), and the gene with the most significant number of amino acids was *AhCASP16* (containing 810 amino acids). The molecular weight of *AhCASPs* was between approximately 7734.14 and 89,355.45 Da, including 17 acidic proteins (pI ≤ 7) and 63 basic proteins (pI ≥ 7). In the *AhCASPs* gene family, 51 *AhCASPs* genes had an instability coefficient of less than 40, which were stable proteins. The instability coefficient of 29 *AhCASPs* genes was greater than 40, which belonged to unstable proteins. The average hydrophilic coefficient of *AhCASP33*, *AhCASP40*, *AhCASP72*, *AhCASP30*, *AhCASP25*, *AhCASP66*, *AhCASP61*, *AhCASP9*, *AhCASP11*, *AhCASP67*, and *AhCASP24* is less than 0, indicating that these proteins exhibit hydrophilic properties. Furthermore, the average hydrophilic coefficient of the 69 *AhCASPs* genes exceeded 0, suggesting that they are classified as non-hydrophilic proteins.

The results of in silico analysis showed that the localization of different *AhCASPs* family members was different: 69.5% of the *AhCASPs* family proteins were located in the cell membrane, 19.5% of the AhCASPs proteins were located in the chloroplast, and 14.6% of the AhCASPs proteins were situated within the nucleus. It is worth noting that *AhCASP6* and *AhCASP52* are located on peroxisomes, *AhCASP22* is located on chloroplasts and Golgi apparatus, and *AhCASP65* is located on cell membranes and Golgi apparatus, indicating that they may play different biological functions. In summary, the results showed that the peanut *AhCASPs* family proteins mainly play biological functions in the cell membrane and adhere to the structural characteristics consistent with membrane proteins.

### 2.2. Chromosome Distribution, Collinearity, and Ka/Ks Analysis

**Figure 1:**
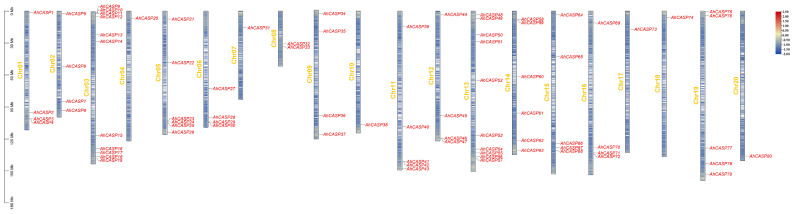
Chromosomal localization of peanut *AhCASPs* family genes. The scale provided represents the chromosome size (Mbp); Chr01–Chr20 represents the name of 20 chromosomes of the peanut genome. The blue and red colors represent low and high gene densities, respectively.

According to the chromosomal localization analysis of 80 *AhCASPs* family genes in peanuts, the distribution map of *AhCASPs* family genes on peanut chromosomes was drawn (Figure 1). The results indicated that *AhCASPs* genes were distributed on each peanut chromosome and distributed on 20 chromosomes. According to the physical location on the chromosome, all *AhCASPs* genes were renamed uniformly. Only one gene was distributed on chromosomes 4, 7, 10, 17, 18, and 20; the remaining genes were mostly distributed on chromosome 3, with eleven genes. A rigorous analysis of gene density reveals that the genes within the three subclusters exhibit a primary concentration at both extremities of the chromosome. Specifically, the gene density is notably higher at the termini, whereas it is comparatively lower in the central region of the chromosome. *AhCASP6*, *AhCASP22*, *AhCASP27*, *AhCASP52*, and the other 23 genes were distributed in the low-density region, with the remaining genes distributed in medium or high-density areas, respectively.

**Figure 2:**
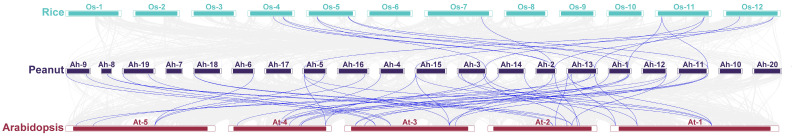
Interspecific collinearity analysis of peanut, *Arabidopsis*, and rice. A grey line represents the collinear block, while a red line highlights the *CASPs* homologous gene pairs.

In addition, the collinear analysis of the genomes between *A. thaliana*, rice, and peanut (Figure 2) revealed that there were 47 linear relationships between 26 genes in *A. thaliana* and 35 genes in peanut; there were 15 linear relationships between 9 genes in rice and 9 genes in peanut. Moreover, 46 peanut *AhCASPs* genes were more conservative, neither collinear with *CASPs* genes in *A. thaliana* nor collinear with *CASPs* genes in rice. Some *CASPs* genes are related to at least two pairs of homologous genes, especially *AhCASP43*, *AhCASP64*, *AhCASP7*, *AhCASP8*, *AhCASP21*, *AhCASP18*, *AhCASP56*, and *AhCASP2*, which are closely related to other genomes and may play an important role in the evolution of *CASPs* gene family. The comparative analysis of *CASPs* genes between peanuts and other plants holds significant importance in establishing interspecific genetic relationships and forecasting gene functionality, thereby contributing to a deeper understanding of genetic diversity and potential applications in plant biology.

The Ka/Ks value is an important parameter for evaluating the evolution of coding sequences and determining the type of selection pressure after repetition [45]. Generally, a Ka/Ks ratio greater than 1, equal to 1, and less than 1 indicates that the gene underwent positive, neutral, and negative or stable selection, respectively [46,47]. In order to understand the selection pattern of *AhCASPs*, we revealed the Ka, Ks, and Ka/Ks ratios of all gene pairs (Table 2). Among the 32 pairs of homologous gene pairs identified, except for *AhCASP2/43*, *AhCASP29/71*, and *AhCASP30/72*, the Ka/Ks values of other 93.75% (30/32) were less than 1, indicating that the *AhCASPs* genes in these groups were subjected to purification selection pressure. The results showed that most *AhCASPs* genes experienced robust purification and selection during evolution, while only a few genes diverged and produced new biological functions. The difference time range of 32 pairs of genome-wide repetitive gene pairs was 0.41~151.82 Mya. Among them, the differentiation time of *AhCASP29/71* in the CASP-b subcluster was the shortest, only 0.41 Mya.

### 2.3. Conserved Motifs, Conserved Domains, and Gene Structure Analysis

**Figure 3:**
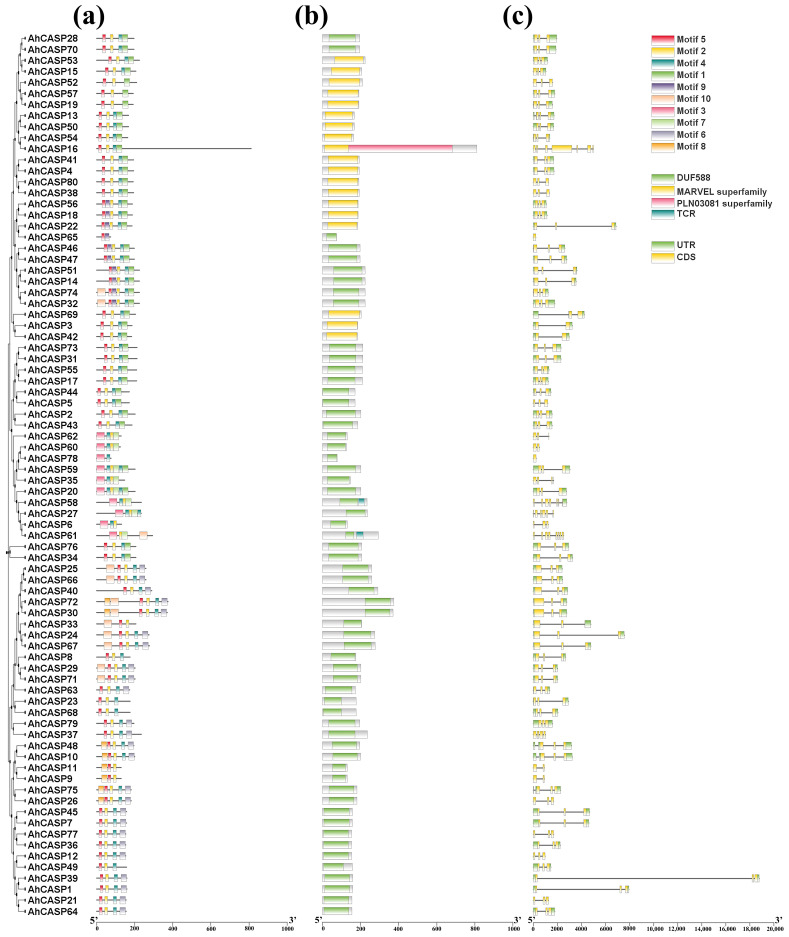
Analysis of conserved motifs, gene domains, and gene structure of peanut *AhCASPs*. Conserved motifs of *AhCASPs* (**a**); the color boxes represent different conserved motifs, as shown in the scheme on the right side of the figure. Conserved domain of *AhCASPs* (**b**): The exon–intron structure of *AhCASPs* (**c**), UTR, and CDS represent untranslated regions and coding sequences, respectively.

The MEME online analysis tool predicted and identified the peanut AhCASPs protein sequence motifs. The statistical results (Figure 3a) showed that 10 motifs were identified from 80 *AhCASPs* family members, namely motifs 1–10. The variation in conserved motifs within each cluster is notable and significant. Although *AhCASP65* contains only one conserved motif, *AhCASP7*8, *AhCASP23*, and *AhCASP68* contain only two motifs, and the number of motifs of the remaining genes is 3–7. It is worth noting that, as shown in Figure 3a, motifs 2, 3 and 10 are widely distributed in the *AhCASPs* family, occupying 80.4%, 95.1%, and 97.6%, respectively, and they are all situated in proximity to the c-terminus of the respective proteins. These findings indicate a high level of conservation among these three motifs within the *AhCASPs* gene family, potentially indicating their significant roles in various standard biological functions. Upon integration of the phylogenetic tree and motif analysis, it is evident that the motif distribution of *AhCASPs* exhibits distinct patterns across different branches. For example, motif 10 is only present in subcluster C; subcluster A lacks motif 6 and motif 9; motif 5 only exists in subcluster B. These results indicate that *AhCASPs* genes with close evolutionary relationships have similar motif compositions.

The analysis results of 80 identified *AhCASPs* protein domains are shown in Figure 3b. *AhCASP16* protein contained the PLN03081 superfamily domain and the MARVEL structure. *AhCASP58* and *AhCASP61* contained the DUF588 structure and TCR domain. The remaining genes contained at least one conserved domain, mainly DUF588 and MARVEL domains. Studies have shown that some scholars have broadened the scope of phylogenetic analysis beyond the plant realm and have uncovered a noteworthy degree of conservation among the CASPs and MARVEL protein families. Notably, these conserved residues are positioned in the transmembrane domains, implying that these specific domains are crucial for the localization of *CASPs* [33].

The intron/exon structure, intron type, and number of genes are typical evolutionary marks of gene families [48]. Therefore, further construction of a protein phylogenetic tree and intron/exon structure map of the peanut *AhCASPs* gene family can further evaluate the structural characteristics of related genes. As shown in Figure 3c, 78 *AhCASPs* members contain 1–8 introns and 2–9 exons, of which *AhCASP65* and *AhCASP78* have no introns.

### 2.4. Phylogenetic Relationship of Peanut AhCASPs

**Figure 4:**
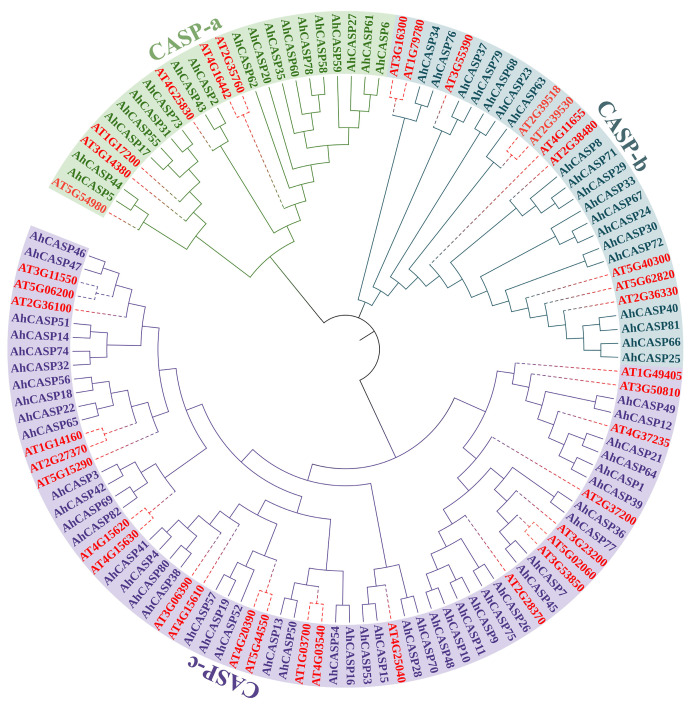
Phylogenetic tree analysis of *CASPs* genes in peanut and *Arabidopsis*. The red dotted line was 39 CASPs proteins in *A. thaliana*; different color branches represent different subclusters.

To comprehensively understand the evolutionary relationships among peanut *AhCASPs* genes, a rigorous multiple sequence alignment analysis was conducted encompassing 80 peanut *AhCASPs* genes and 39 *AtCASPs* genes derived from the model plant *A. thaliana*. Subsequently, phylogenetic trees were generated with the alignment results (Figure 4). According to the genetic relationship, 119 *CASPs* genes were divided into CASP-a, CASP-b, and CASP-c sub-clusters. Among them, the CASP-c subcluster was the most distributed, with a total of 23 *AtCASPs* genes and 44 *AhCASPs* genes. A total of 6 *AtCASPs* genes and 18 *AhCASPs* genes were distributed in the CASP-a subcluster. There were 10 *AtCASPs* genes and 18 *AhCASPs* genes distributed in the CASP-b subcluster. Prior investigations have revealed a definitive association between the *AT2G36100* gene (*AtCASP1*) and salt tolerance in rice [49]. Furthermore, the *AT3G55390* gene (*AtCASPL4C1*) has been documented to be triggered by low-temperature conditions and exerts a negative regulatory effect on plant growth [50]. Additionally, the *AT4G03540* gene (*AtCASPL1C1*) constructs an extracellular barrier, thereby effectively augmenting the salt tolerance capability of sweet sorghum [43]. Therefore, it is speculated that the highly homologous *AhCASPs* genes may possess analogous functionalities. So far, the specific biological function of the peanut *AhCASPs* genes remains unclarified, but the function of some *CASPs* genes in the model plant *A. thaliana* has been verified. A class of *CASPs* proteins with similar evolutionary relationships have identical structures and often play similar biological functions. Therefore, the biological function of the peanut *AhCASPs* gene can be speculated and verified according to the clustering analysis results of peanut and model plant *A. thaliana* CASPs protein.

### 2.5. Prediction of Cis-Acting Elements of AhCASPs Promoter

**Figure 5:**
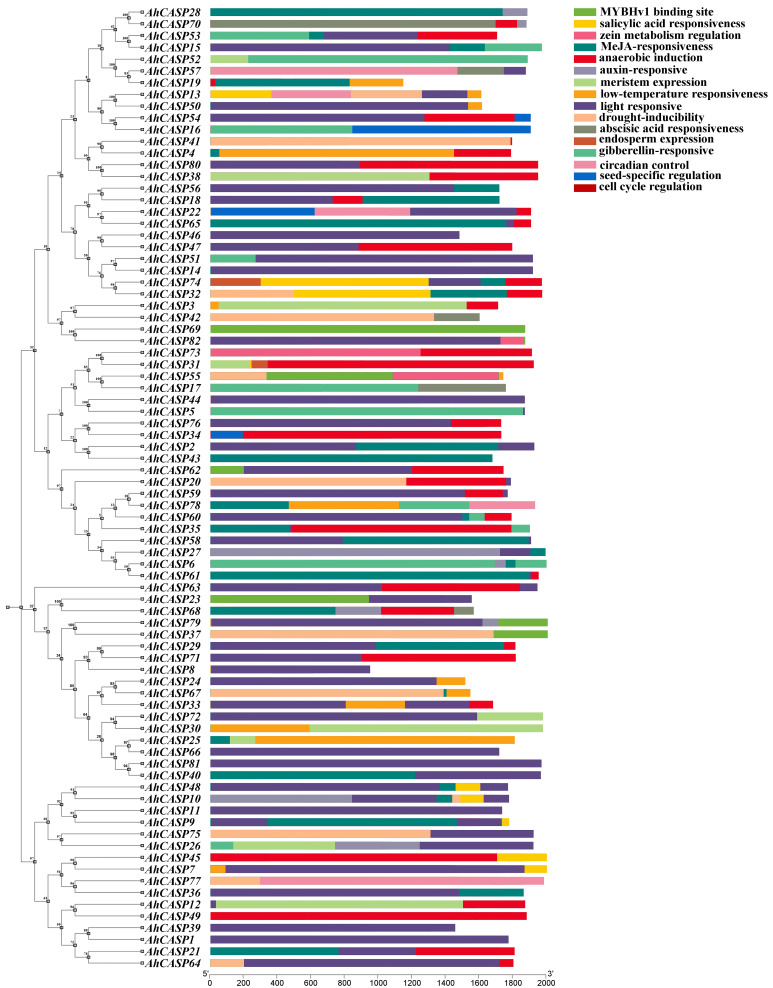
*Cis-*acting elements of peanut *AhCASPs* family members. The upstream sequence of *AhCASPs* gene promoter is 2000 bp and encompasses a diverse array of *cis-*acting elements, encompassing elements responsive to light, hormones, drought conditions, low temperatures, anaerobic environments, and wounding, in addition to specific elements associated with meristems, seeds, and endosperms.

**Figure 6:**
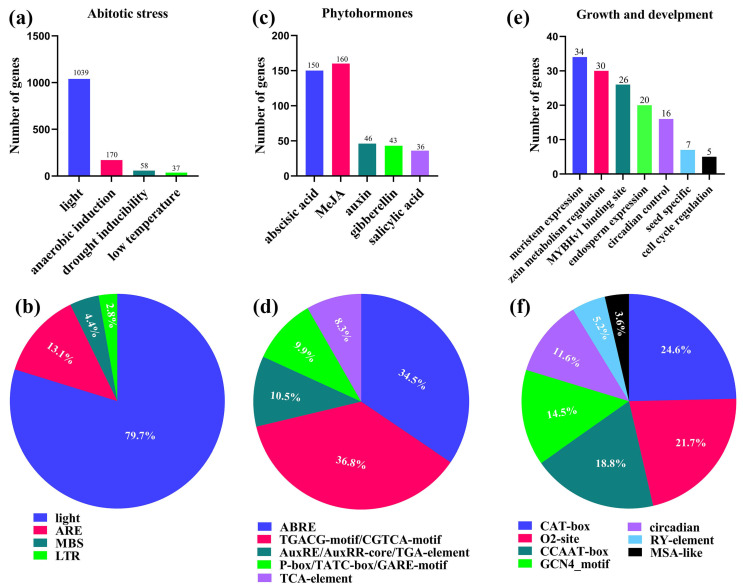
Regarding the *cis-*regulatory elements in the *AhCASPs* promoter region, detailed statistical and analytical results are as follows: (**a**,**c**,**e**) the total number of *AhCASPs* genes involved in abiotic stresses, phytohormones, and *cis-*element growth and developmental categories has been counted. Specifically, the (**b**,**d**,**f**) pie charts show the percentage proportion of several *cis-*elements in each category, including (**b**) abiotic stress response, (**d**) phytohormone response, and (**f**) plant growth and development response. Different colors represent different *cis-*acting elements and their proportions in the *AhCASPs* gene.

*Cis-*acting elements constitute a specific category of DNA sequences located in the initiation region of gene transcription, which play a key role in regulating gene transcription. They promote or inhibit gene transcription by binding to transcription factors [51,52]. The promoter sequences of 2000 bp upstream of ATG of the 80 *AhCASPs* CDS was extracted and analyzed. After filtering out unknown and untrustworthy elements, a total of three types of *cis-*acting elements were identified, including abiotic stress response, plant hormone response, and growth and development response elements (Figure 5 and Figure 6). Four abiotic stress response elements have been identified: light, anaerobic, drought, and low temperature. Among these elements, the following motifs have been detected: I-box, ATCT, Box 4, GT1, GA (accounting for 79.7% in light response), ARE (13%), MBS (4.4%), and LTR (2.8%). Among them, most are light response elements, suggesting that the *AhCASPs* gene family may play a role in regulating plant photomorphogenesis. There were five types of response elements related to hormone regulation (gibberellin (GA), methyl jasmonate (MeJA), SA, auxin (IAA), and ABA, including ABRE (34.5%), CGTCA-motif/TGACG-motif (36.8%), AuxRE/AuxRR-core/TGA-element (10.6%), P-box/TATC-box/GARE-motif (9.9%) and TCA-element (8.3%)). Among them, ABRE and CGTCA-motif/TGACG-motif are the most prominent types, accounting for 34.5% and 36.8% of all identified *cis-*acting hormone elements. In addition, after in-depth research, we identified seven growth and developmental response elements, including endosperm expression, meristem expression, circadian control, zein metabolism, cell cycle regulation, seed specificity, and MYBHv1 binding sites. These crucial components encompass CAT-box (24.6%), O2-site (21.7%), CCAAT-box (18.8%), GCN4_motif (14.5%), circadian (11.6%), RY-element (5.1%), and MSA-like (3.6%). It is surmised that the *AhCASPs* family is potentially implicated in diverse developmental processes and has a significant role in hormone regulation and stress response mechanisms.

### 2.6. miRNA Target Gene Prediction and GO Enrichment Analysis

**Figure 7:**
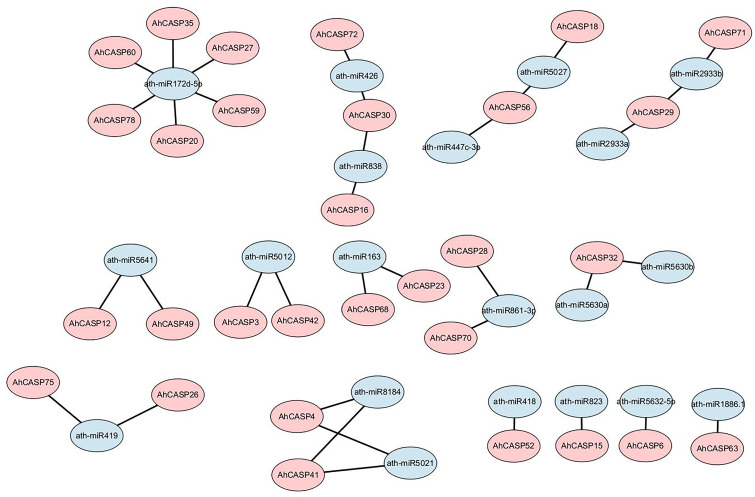
Predicted miRNAs that may target *AhCASPs*. The pink shape corresponds to the *AhCASPs* gene, and the blue represents the indicated miRNA.

The psRNATarget service has been utilized to examine the regulatory mechanisms of miRNAs with the expression of the *AhCASPs* gene. In order to better study how miRNAs regulate *AhCASPs* genes, we identified 20 different miRNAs for 37 different *AhCASPs* genes (Figure 7). The results showed that Ath-miR172d-5p targeted the most genes. Twelve miRNAs, including ath-miR2933a, ath-miR2933b, ath-miR861-3p, ath-miR163, ath-miR419, ath-miR426, ath-miR5012, ath-miR5021, ath-miR5027, ath-miR5641, ath-miR8184, ath-miR838, each targeted two different genes, respectively. Seven miRNAs including ath-miR418, ath-miR447c-3p, ath-miR5630a, and ath-miR5630b only regulate one gene, *AhCASP52*, *AhCASP56*, *AhCASP32*, *AhCASP32*, *AhCASP6*, *AhCASP63*, and *AhCASP15*. Seven genes, including *AhCASP29*, *AhCASP56*, and *AhCASP32*, were targeted by more than one miRNA.

**Figure 8:**
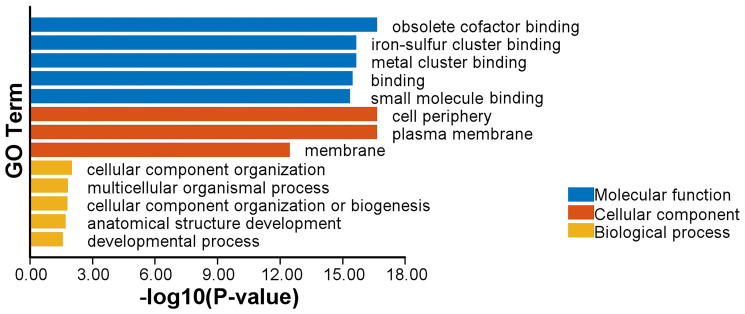
Highly enriched GO annotations in MF, CC, and BP classifications of *AhCASPs* genes.

In order to gain a deeper understanding of the molecular-level function of *AhCASPs* genes, we conducted a GO enrichment analysis encompassing 80 *AhCASPs* (Figure 8). GO enrichment analysis can be divided into three categories: molecular function (MF), cellular component (CC) and biological process (BP). At the molecular function level, obsolete cofactor binding (GO: 0048037) as well as iron–sulfur cluster binding (GO: 0051536), metal cluster binding (GO: 0051540), binding (GO: 0005488), small molecule binding (GO: 0036094) and other processes were significantly enriched. At the cellular component level, the highly enriched items were the cell periphery (GO: 0071944), plasma membrane (GO: 0005886), and membrane (GO: 0016020). At the level of biological processes, the highly enriched terms were cellular component organization (GO: 0016043), multicellular organismal process (GO: 0032501), cellular component organization or biogenesis (GO: 0071840), anatomical structure development (GO: 0048856), developmental process (GO: 0032502), etc.

### 2.7. Differential Expression Analysis of AhCASPs Genes

**Figure 9:**
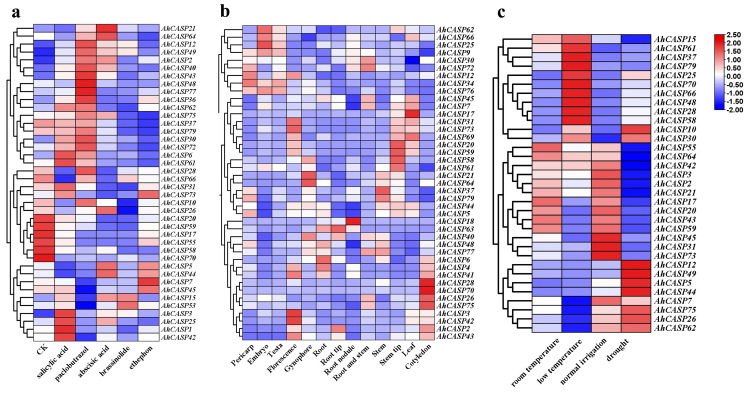
Heatmap of expression patterns of peanut *AhCASPs* based on FPKM values for different hormones (**a**), different organs (**b**), and different treatments (**c**). TBtools was used to perform log2 transformation and visualization of FPKM values. The upper right scale represents the level of gene expression.

According to rigorous studies, it has been established that numerous plant hormones play a pivotal role in the response to abiotic stress. They serve as intermediaries in the plant’s adaptive response to such stress conditions, thereby safeguarding the plants from the adverse impacts of abiotic stress [53]. In the analysis of *cis-*acting elements, we found that the *AhCASPs* promoter region has many elements that respond to stress and various plant hormone signals, indicating that the *AhCASPs* gene is postulated to contribute significantly to plants’ stress regulation response mechanisms. In order to delve deeper into the functional role of *AhCASPs* family genes in adverse stress conditions, we conducted a comprehensive analysis of the transcriptional expression patterns of the *AhCASPs* gene family, utilizing transcriptome data derived from cultivated peanuts under low-temperature and drought stress, as well as exposure to various hormone treatments. In order to understand the expression changes of *AhCASPs* under different hormones, the FPKM value of transcriptome data was used to screen and construct the response heat map of 40 *AhCASPs* genes to five stress-related plant growth regulators (SA, paclobutrazol, ABA, brassinolide, and ethylene) (Figure 9a). After SA treatment, compared with the control group and other hormone treatments, the expression levels of *AhCASP3*, *AhCASP25*, *AhCASP1*, *AhCASP42*, *AhCASP6*, and *AhCASP61* genes were significantly increased, and the expression levels of *AhCASP66*, *AhCASP5*, and *AhCASP44* genes were down-regulated. The response of *AhCASPs* genes to paclobutrazol was divided into two types. In the first type, the expression levels of 16 genes were up-regulated, while in the second type, except for *AhCASP28* and *AhCASP10*, the expression levels of other genes were down-regulated. Following ABA treatment, a comparative analysis was conducted against the control group and alternative hormone treatments, revealing distinct variations in the gene expression levels of the target subjects. *AhCASP21*, *AhCASP64*, *AhCASP75*, *AhCASP26*, *AhCASP5*, *AhCASP44*, and *AhCASP15* were significantly up-regulated, and the expression levels of the other 33 genes were down-regulated. After brassinolide treatment, the expression levels of *AhCASP15*, *AhCASP53*, *AhCASP12*, and *AhCASP49* genes were significantly up-regulated, and the expression levels of 36 genes such as *AhCASP62*, *AhCASP75*, *AhCASP10*, and *AhCASP26* were down-regulated. Following ethylene treatment, in comparison to the control group and alternative hormonal interventions, the transcriptional levels of *AhCASP73* and *AhCASP7* genes were observed to increase, while the expression levels of the remaining 38 genes underwent a decrease.

To investigate the different expression patterns and tissue expression specificity of *AhCASPs* family genes, we used the FPKM value of transcriptome data to screen and analyze the expression levels of 63 *AhCASPs* genes in 13 organs of cultivated peanut (including pericarp, embryo, testa, florescence, gynophore, root, root tip, root nodule, root and stem, stem, stem tip, leaf, and cotyledon). We used TBtools-II software to draw an expression heat map of these organs (Figure 9b). The findings indicate that distinct expression patterns of various genes exist across different organs. Among them, *AhCASP13* and *AhCASP50* were highly expressed in the seed coat; the expression levels of *AhCASP25*, *AhCASP39*, and *AhCASP53* were the highest in embryos. *AhCASP73*, *AhCASP17*, and *AhCASP31* had higher expression levels in leaves. The expression levels of *AhCASP6*, *AhCASP41*, and *AhCASP4* were higher in roots, flowering, and cotyledons, and the expression levels of these genes were lower in other organs. It is hypothesized that various *AhCASPs* genes may solely exhibit biological functions in designated organs. In addition, the response of different *AhCASPs* to low temperature and drought treatment was not the same. After low temperature treatment (Figure 9c), the expression levels of 21 genes, such as *AhCASP15*, were up-regulated, and 12 genes, such as *AhCASP26*, were down-regulated. The expression trend of 11 genes, such as *AhCASP12*, *AhCASP49*, *AhCASP5*, and *AhCASP44*, under drought treatment was notably elevated compared to the control group (normal irrigation conditions). The expression levels of *AhCASP15*, *AhCASP53*, *AhCASP21*, *AhCASP64*, *AhCASP31*, *AhCASP3*, and *AhCASP42* were significantly down-regulated. Notably, the expression levels of *AhCASP11*, *AhCASP55*, *AhCASP70*, and *AhCASP62* were significantly diminished under conditions of low temperature and drought, respectively. This observation suggests these four genes are likely unrelated to the adaptive response to this environmental stress but may play a key role in other biological processes.

## 3. Discussion

*CASPs* are membrane proteins that are exclusively found in the Casparian strip. This gene is important in regulating plant growth and development and stress response mechanisms, as it is a critical component in forming and controlling the Casparian strip [33,38]. Several plant species, including *A. thaliana* (39 members) [33], rice and cotton (19 and 48 members, respectively) [39,40], and patchouli (156 members) [42], have been identified as containing this gene family. However, as an important oil crop, the identification and analysis of the peanut *AhCASPs* gene family have not been reported. Moreover, they exhibit vulnerability to diverse abiotic stresses, encompassing phenomena such as hypothermic conditions and drought. Therefore, identifying and analyzing the response of peanut *AhCASPs* genes to various abiotic stresses is of great significance. In this study, a total of 80 *AhCASPs* genes were identified in the peanut genome and divided into 3 sub-clusters along with *Arabidopsis CASPs* genes (Figure 4). Compared with *Arabidopsis* and rice, the number of *AhCASPs* genes in peanuts increased significantly, which may be because the cultivated peanut is an allotetraploid (AABB type genome; 2n = 4x = 40) [54,55,56]. Several variables determine the number of gene family members in different species, including natural selection, genome doubling time, and tandem duplication [57]. Previous research has demonstrated that genes frequently endure tandem repeat events during evolution to enhance the number of gene family members [46]. Simultaneously, gene replication can induce functional differentiation among genes and expedite the emergence of new genes [58]. This study showed that 32 gene pairs in the peanut *AhCASPs* gene family had a Ka/Ks ratio < 1, suggesting that the *AhCASPs* gene may be retained to promote the amplification of *CASPs* genes in peanuts and may be subject to purification selection pressure. Unsurprisingly, the two genes in each pair are from the same subfamily, which strongly implies that *CASPs* genes are more conserved within the same subfamily.

Transcriptional regulation is the primary mechanism of gene expression regulation in eukaryotes. *Cis* elements are critical components in regulating gene transcription, and they play a critical role in various biological processes, including hormonal responses, abiotic stress reactions, and developmental trajectories [59]. The auxin-induced promoters are typically the source of the *cis-*acting elements that contain the AuxRE and DR5-motif [60]. Light-induced promoters act as an important regulatory sequence, usually containing core elements such as G-box, rich in AT, GT1-motif, and I-box [61]. Promoters that harbor CATGTG and CACG *cis-*acting elements are responsive to drought stress conditions [62]. In plants, IAA (indole-3-acetic acid), GA (gibberellic acid), SA (salicylic acid), ABA (abscisic acid), and MeJA (methyl jasmonate) are essential for the regulation of plant growth, ensuring the proper development and functioning of the plant’s various biological development, processes, and stress adaptation mechanisms [63,64]. The peanut genome was used to extract the promoter sequences of 2000 bp upstream of ATG of the 80 *AhCASPs* CDS, and their *cis-*acting elements were identified in this study. The *cis-*acting elements associated with stress and hormones were the primary focus of our analysis (Figure 5 and Figure 6). The findings indicated that the *cis-*acting elements associated with hormone response were ABA, GA, IAA, MeJA, and SA. Transcriptome data analysis showed that ABA and SA treatments had different induction effects on *AhCASPs* genes. Various hormones play significant roles in the regulatory mechanism of *CASPs*, especially ABA and MeJA hormones, which occupy more central and critical positions in the hormonal regulatory system [65]. Additionally, we have identified *cis-*acting elements that are associated with the stress response. These elements primarily respond to environmental stresses like drought and low temperature. The close proportion of *cis-*acting elements in different species and hypoxia suggests that families of *CASPs* may have a similar regulation pattern in peanuts. The *CASPs* family may be involved, collectively, in the control of several hormonal and stress responses to *cis-*acting elements that improve plant tolerance to the external environment, allow plants to react to biotic and abiotic stresses, and help to understand the complex mechanisms underlying *cis-*acting regulatory components in the stress response process.

It has previously been reported that miRNAs are responsive to non-biological stress [66], suggesting that these miRNA families hold significant potential in regulating biological processes associated with stress-related responses. Particular miRNA families are well-established in their roles in the developmental progression of plants and their adaptive responses to abiotic stresses [67,68]. Previous studies have documented the induction of miR861 in *A. thaliana* under high light conditions [69]. Furthermore, miRNAs such as ath-miR2933a, ath-miR2933b, and ath-miR5021 have been identified as key regulators in the calcium signaling pathway, mediating the regulation of calcium response genes during drought stress [70]. miR408 and ath-miR5632-5p are implicated in diverse abiotic stress responses, including drought [71,72]. Additionally, miRNA172, which inhibits the *AP2* gene, has been reported to regulate flower development and flowering time across various plant species, including *A. thaliana*, barley, soybean, and rice [73,74,75,76]. In turmeric, miR5021 participates in the biosynthesis of terpenoids and isoquinoline alkaloids [77]. Our research has identified a total of 20 distinct miRNAs targeting 37 different *AhCASPs* genes (Figure 7). The identified differential expression patterns of *AhCASPs* genes have been observed to exhibit a correlation with stress-responsive miRNAs that specifically target these genes. This correlation underscores the significance of *AhCASPs* in the adaptation to stress conditions and plant developmental processes.

The stress and hormone transcriptional expression analysis indicated that the transcriptional expression patterns of *AhCASPs* under stress could be divided into high and medium–low expressions. Notably, the expression levels of *AhCASP55*, *AhCASP73*, *AhCASP48*, *AhCASP31*, *AhCASP42*, *AhCASP3*, and *AhCASP41* under low-temperature stress and the expression levels of *AhCASP3*, *AhCASP31*, *AhCASP73*, *AhCASP44*, *AhCASP62*, *AhCASP5*, *AhCASP49*, and *AhCASP12* under drought stress were high. These genes contained low-temperature- and drought-response elements (Figure 5). This indicates that these genes may respond to low temperature or drought stress. Previous studies have found that *Arabidopsis AT3G55390* (*AtCASPL4C1*) is crucial in resisting low-temperature stress conditions [50]. Some members of banana *MaCASPs* are specifically expressed under low-temperature induction [41]. In addition, *AhCASP76*, *AhCASP50*, *AhCASP54*, and *AhCASP76*, *AhCASP71*, *AhCASP50*, *AhCASP79*, *AhCASP1*, *AhCASP39*, *AhCASP69*, *AhCASP11*, *AhCASP53*, and other genes were down-regulated under cold and drought stress, respectively. Therefore, how and what role different *AhCASPs* play in different stress conditions remains to be further verified.

## 4. Material and Methods

### 4.1. Identification of AhCASPs Genes

The *Arabidopsis* protein sequence and genome files were obtained from the TAIR website (https://www.arabidopsis.org/ accessed on 2 March 2024), and the peanut genome and gene annotation files were downloaded from the peanut website (http://peanutgr.fafu.edu.cn/ accessed on 2 March 2024). Two distinct methodologies were employed to search for and identify members of the peanut *AhCASPs* family. Firstly, 39 protein sequences downloaded from the *Arabidopsis* website were used as reference sequences for blast alignment [78] to obtain candidate *AhCASPs* family members. Subsequently, a search was conducted on the Pfam website [79] for the Pfam identifier (PF04535) on the *AhCASPs* family domain, and the respective Hidden Markov Model (HMM) was then procured for further analysis. The Simple HMM search online program [80] was utilized to initially screen protein sequences analogous to the hidden Markov model domain, applying a stringent e-value threshold of less than 10^−5^ to identify potential candidate members. They were submitted to CDD (https://www.ncbi.nlm.nih.gov/CDD accessed on 13 March 2024) and SMART (http://smart.embl.de/ accessed on 8 March 2024); to ensure the accuracy of the domain, it is necessary to verify it and subsequently eliminate any sequences that do not include the specified domain. The ProtParam (https://web.expasy.org/protparam/ accessed on 22 March 2024) online program [81] was employed to forecast its physicochemical properties, including relative molecular weight (Mw), amino acid number, hydrophilic large average (GRAVY) and isoelectric point (pI). The Plant-mPLoc 2.0 online software (http://www.csbio.sjtu.edu.cn/bioinf/plant-multi/, accessed on 11 April 2024) was used to predict the in silico analysis of *AhCASPs* [82].

### 4.2. Chromosome Distribution, Collinearity, and Ka/Ks Analysis

The Ka/Ks values for both repetitive genes and tandem repeats were precisely computed using the KaKs_Calculator 2.0 online program [83]. The peanut’s differentiation time (Mya) was calculated using the formula T = Ks/2r. Based on prior research, the value of r, representing the neutral substitution rate, was established at 8.5 × 10^−9^ [55].

### 4.3. Conserved Motifs, Domains, and Gene Structure Analysis

The positional data on exons, introns, and untranslated regions (UTRs) of the *AhCASPs* family genes were retrieved from the peanut genome annotation file (gff), and the structural representation of *AhCASPs* genes was subsequently generated utilizing TBtools-II software (version 2.085). The conserved motifs of *AhCASPs* were analyzed using MEME online (http://meme-suite.org/, accessed on 12 April 2024). The maximal number of motifs is 10, and the length ranges from 6 to 50 amino acids [84]. The conserved domains were inputted into the AhCASPs protein sequence using the online tool Batch CD-Search to generate the corresponding domain files [85]. Visual analysis was conducted using TBtools-II [86].

### 4.4. Phylogenetic Relationship of Peanut AhCASPs

MEGA 11 software was employed to conduct multiple sequence alignments on the full-length *Arabidopsis* and peanut *CASPs* sequence*s* to investigate the phylogenetic relationship of peanut *AhCASPs* family members [87]. The NJ method was employed to generate the phylogenetic tree, which was then used to create the comparison results. The phylogenetic tree’s bootstrap method was configured to 1000, and all other parameters were set to their default values. Ultimately, the tree was colored using iTOL (https://itol.embl.de/, accessed on 24 April 2024) [88].

### 4.5. Prediction of Cis-Acting Elements of AhCASPs Promoter

The 2000 base pair region upstream of the *AhCASPs* start codon (ATG) was successfully extracted, and the *cis-*acting elements in this promoter were predicted and analyzed using PlantCARE software (https://bioinformatics.psb.ugent.be/webtools/plantcare/html/, accessed on 14 April 2024) [89]. In addition, *cis-*acting elements were visualized using the TBtools-II software.

### 4.6. miRNA Target Gene Prediction and GO Enrichment Analysis

We used the eggNOG-mapper online tool (http://eggnog-mapper.embl.de/, accessed on 8 April 2024) to perform a detailed functional annotation of the peanut genome file [90]. This analysis method can effectively reveal the molecular function of genes, biological processes, and their complex association with cellular components. In addition, we also used GO enrichment analysis to accurately label related metabolic pathways, thus providing a crucial reference for deepening our comprehension of these genes’ role in specific metabolic processes. By combining the comprehensive analysis of eggNOG-mapper and TBtools-II, we can more fully understand the functional characteristics of the identified genes and their specific roles in the metabolic process.

In order to identify miRNAs that target the transcripts of *AhCASPs* family members, the coding sequence (CDS) was submitted to psRNATarget (https://www.zhaolab.org/psRNATarget/, accessed on 24 April 2024) to set the following parameters: (1) the expected value was 3; (2) Max UPE was 25 and flank length upstream was 17 NT; and the downstream was 13 NT to find the target of miRNA and analyze the targeting relationship of miRNA-*AhCASPs* [91]. Finally, the Cytoscape software (version 3.10.2) was used to visualize the target relationship network [92].

### 4.7. Differential Expression Analysis of AhCASPs Genes

Transcriptome data (http://peanutgr.fafu.edu.cn accessed on 10 May 2024) were used to extract the expression data of all *AhCASPs* in different stress conditions (blank, drought, and low temperature), different plant growth regulators (ABA, brassinolide, ethephon, paclobutrazol, and SA) and different tissues and organs (root, stem, leaf, peel, embryo, and other 13 organs) [93]. Transcriptome data of *AhCASPs* genes were screened from the above data, and preliminary screening of these data was performed using Excel 2019. Then, TBtools-II was used to reprocess the data and make heat maps for visualization.

## 5. Conclusions

Peanuts are an important economic and oil crop, providing oil and protein for human nutrition. During the growth phase, peanut production is subject to various adverse stress conditions, including extreme temperatures, elevated salinity, and drought. Many peanut gene families have been reported with the establishment of the peanut genome database, but our understanding of the peanut *AhCASPs* gene family is minimal. Based on their phylogenetic tree connection, the 80 peanut *AhCASPs* genes were identified in this research and grouped into three subclusters. Sequence analysis showed that most AhCASPs proteins contained conserved MAVEL and DUF588 domains. Apart from *AhCASP2*/*43*, *AhCASP29/71*, and *AhCASP30/72*, of the 32 pairings of homologous gene pairs found, 93.75% (30/32) of the other pairs had Ka/Ks values < 1, suggesting that purification selection pressure was acting on the *AhCASPs* genes in these groups. The analysis of *cis-*acting elements revealed that 80 *AhCASPs* genes harbored many hormone-regulatory, light-responsive, and environmental-stress-associated elements. Thus, to explore the many functions of the members of the *AhCASPs* family in peanuts, future studies should concentrate on the functional identification of *AhCASPs* genes. The findings of this work provide significant new information on the evolution of the *AhCASPs* gene family in peanuts and provide the theoretical groundwork for further research on the stress-responsive function of *AhCASPs* and molecular processes of *CASPs* genes in other species.

## Figures and Tables

**Table 1:** Physicochemical properties and in silico analysis of peanut *AhCASPs*.

Gene ID	Gene Name	Length	Molecular Weight (Da)	pI	Instability Index	Aliphatic Index	GRAVY	Subcellular
AH01G00870.1	*AhCASP1*	158	17,410.640	5.030	32.870	108.040	0.946	Cell membrane
AH01G22790.1	*AhCASP2*	200	22,455.880	9.510	34.390	101.000	0.495	Chloroplast
AH01G27590.1	*AhCASP3*	184	19,521.210	9.860	30.550	125.000	0.726	Chloroplast
AH01G27600.1	*AhCASP4*	192	20,653.250	9.550	29.280	114.740	0.617	Cell membrane
AH02G02150.1	*AhCASP5*	170	18,992.310	8.240	33.890	110.120	0.680	Cell membrane
AH02G14400.1	*AhCASP6*	129	14,159.910	10.530	32.350	123.800	0.561	Peroxisome
AH02G19940.1	*AhCASP7*	155	17,076.360	9.250	42.090	99.480	0.751	Cell membrane
AH02G23610.1	*AhCASP8*	174	19,302.090	6.720	25.710	92.590	0.217	Cell membrane/Chloroplast/Peroxisome
AH03G00710.1	*AhCASP9*	128	13,763.520	6.690	53.330	89.920	−0.098	Cell membrane
AH03G01440.1	*AhCASP10*	199	20,893.680	5.800	45.110	89.750	0.261	Cell membrane
AH03G01480.1	*AhCASP11*	128	13,763.520	6.690	53.330	89.920	−0.098	Cell membrane
AH03G04600.1	*AhCASP12*	152	16,577.660	6.680	29.900	120.590	0.968	Cell membrane
AH03G14800.1	*AhCASP13*	166	18,174.900	9.610	21.420	127.530	0.887	Cell membrane/Nucleus
AH03G17620.1	*AhCASP14*	222	23,645.640	9.020	33.690	101.580	0.539	Cell membrane
AH03G30890.1	*AhCASP15*	205	22,831.880	8.090	28.810	88.540	0.477	Cell membrane
AH03G36880.1	*AhCASP16*	810	89,355.450	8.830	42.510	100.300	0.272	Chloroplast
AH03G39480.1	*AhCASP17*	209	22,590.160	7.570	48.110	92.060	0.412	Cell membrane
AH03G42570.1	*AhCASP18*	187	20,528.560	10.150	43.820	115.240	0.811	Chloroplast
AH03G44420.1	*AhCASP19*	191	20,647.480	9.610	24.700	111.360	0.536	Chloroplast/Nucleus/Peroxisome
AH04G05340.1	*AhCASP20*	201	21,647.660	9.240	26.370	112.440	0.674	Cell membrane/Chloroplast/Golgi apparatus/Peroxisome
AH05G06060.1	*AhCASP21*	153	16,646.660	4.460	29.570	110.330	1.061	Cell membrane
AH05G18110.1	*AhCASP22*	184	19,606.320	9.370	26.220	129.460	0.930	Chloroplast/Golgi apparatus
AH05G29740.1	*AhCASP23*	175	18,952.240	9.550	28.090	115.260	0.704	Chloroplast
AH05G30620.1	*AhCASP24*	273	29,920.480	8.790	47.580	89.300	−0.052	Chloroplast/Nucleus
AH05G31520.1	*AhCASP25*	258	28,998.780	7.710	63.510	66.940	−0.307	Nucleus
AH05G37160.1	*AhCASP26*	180	19,143.040	7.710	46.220	89.060	0.441	Cell membrane
AH06G18360.1	*AhCASP27*	234	26,676.070	9.910	28.460	111.200	0.496	Chloroplast
AH06G24120.1	*AhCASP28*	194	21,650.230	8.350	45.760	100.050	0.537	Cell membrane
AH06G25820.1	*AhCASP29*	200	22,063.660	9.390	45.660	100.600	0.149	Nucleus
AH06G26150.1	*AhCASP30*	370	40,467.550	7.750	61.550	72.190	−0.324	Nucleus
AH07G11350.1	*AhCASP31*	211	22,581.910	5.880	24.940	87.010	0.363	Cell membrane
AH08G15760.1	*AhCASP32*	222	23,721.760	8.910	34.200	100.270	0.586	Cell membrane/Nucleus
AH08G17980.1	*AhCASP33*	205	22,704.030	8.980	57.460	75.120	−0.439	Nucleus
AH09G03020.1	*AhCASP34*	205	22,248.690	9.580	49.210	93.800	0.290	Nucleus
AH09G11490.1	*AhCASP35*	145	15,417.410	9.870	26.260	126.970	0.806	Cell membrane/Chloroplast/Golgi apparatus/Peroxisome
AH09G21610.1	*AhCASP36*	152	16,448.460	7.750	32.140	115.660	0.892	Cell membrane
AH09G31200.1	*AhCASP37*	234	25,999.460	6.780	40.500	84.620	0.213	Cell membrane
AH10G25080.1	*AhCASP38*	192	20,478.190	9.280	21.540	123.910	0.748	Cell membrane
AH11G08640.1	*AhCASP39*	158	17,376.620	5.030	34.090	110.510	0.953	Cell membrane
AH11G21400.1	*AhCASP40*	289	31,997.860	7.160	60.850	64.500	−0.383	Nucleus
AH11G30050.1	*AhCASP41*	192	20,653.250	9.550	29.280	114.740	0.617	Cell membrane
AH11G30070.1	*AhCASP42*	183	19,441.120	9.860	33.650	124.100	0.745	Cell membrane/Chloroplast/Golgi apparatus/Nucleus/Peroxisome
AH11G35090.1	*AhCASP43*	185	20,578.650	9.490	30.560	106.000	0.487	Chloroplast/Nucleus
AH12G02290.1	*AhCASP44*	170	18,992.310	8.240	33.890	110.120	0.680	Cell membrane
AH12G22250.1	*AhCASP45*	155	17,076.360	9.250	42.090	99.480	0.751	Cell membrane
AH12G35210.1	*AhCASP46*	197	21,076.530	6.390	20.650	113.400	0.674	Cell membrane
AH12G35220.1	*AhCASP47*	197	20,858.530	8.620	32.210	114.010	0.827	Cell membrane
AH13G03220.1	*AhCASP48*	195	20,666.460	5.400	43.300	91.590	0.274	Cell membrane
AH13G06630.1	*AhCASP49*	156	16,936.820	5.610	37.610	110.580	0.855	Cell membrane
AH13G17400.1	*AhCASP50*	166	18,128.810	9.610	20.910	128.670	0.887	Cell membrane
AH13G20190.1	*AhCASP51*	222	23,717.800	9.040	37.090	103.740	0.584	Cell membrane
AH13G29210.1	*AhCASP52*	208	22,435.490	9.600	30.120	113.560	0.447	Peroxisome
AH13G34990.1	*AhCASP53*	222	24,705.100	8.090	34.410	90.540	0.441	Cell membrane
AH13G40110.1	*AhCASP54*	161	17,258.670	9.640	33.040	138.140	0.988	Cell membrane/Nucleus
AH13G42370.1	*AhCASP55*	209	22,589.130	7.570	46.280	91.580	0.401	Cell membrane
AH13G45190.1	*AhCASP56*	187	20,521.580	10.150	38.000	114.170	0.831	Chloroplast
AH13G47090.1	*AhCASP57*	191	20,643.480	9.610	22.840	113.930	0.548	Chloroplast/Nucleus/Peroxisome
AH14G06350.1	*AhCASP58*	233	26,029.940	9.630	33.820	85.280	0.194	Cell membrane/Cell wall/Chloroplast/Mitochondrion/Nucleus/Peroxisome
AH14G06890.1	*AhCASP59*	201	21,680.650	9.240	25.410	109.550	0.644	Cell membrane/Chloroplast/Golgi apparatus/Peroxisome
AH14G18560.1	*AhCASP60*	124	13,547.170	9.370	22.580	128.790	0.841	Cell membrane
AH14G22800.1	*AhCASP61*	292	32,598.040	9.510	48.550	73.420	−0.216	Cell membrane/Mitochondrion/Nucleus
AH14G32740.1	*AhCASP62*	128	13,859.640	9.230	14.940	137.730	0.930	Cell membrane/Chloroplast
AH14G40830.1	*AhCASP63*	171	18,650.630	6.260	25.350	105.500	0.645	Cell membrane
AH15G02130.1	*AhCASP64*	153	16,630.660	4.460	29.570	110.980	1.078	Cell membrane
AH15G14530.1	*AhCASP65*	72	7734.140	8.340	36.500	105.830	0.590	Cell membrane/Golgi apparatus
AH15G21900.1	*AhCASP66*	258	28,924.680	7.710	62.470	67.710	−0.304	Nucleus
AH15G23250.1	*AhCASP67*	275	30,269.910	8.900	46.840	87.240	−0.077	Chloroplast
AH15G24290.1	*AhCASP68*	175	18,998.330	9.550	31.870	114.170	0.699	Chloroplast
AH16G06190.1	*AhCASP69*	202	22,036.010	9.270	35.040	108.560	0.648	Chloroplast
AH16G29800.1	*AhCASP70*	194	21,654.240	8.350	45.610	97.530	0.512	Cell membrane
AH16G32140.1	*AhCASP71*	200	22,098.510	8.520	46.370	98.150	0.111	Nucleus
AH16G32610.1	*AhCASP72*	373	40,707.730	7.130	60.930	71.610	−0.351	Nucleus
AH17G10700.1	*AhCASP73*	211	22,581.910	5.880	24.940	87.010	0.363	Cell membrane
AH18G06070.1	*AhCASP74*	222	23,731.800	8.910	33.460	100.720	0.586	Nucleus
AH19G00710.1	*AhCASP75*	180	19,143.040	7.710	46.220	89.060	0.441	Cell membrane
AH19G04550.1	*AhCASP76*	205	22,248.690	9.580	49.210	93.800	0.290	Nucleus
AH19G27380.1	*AhCASP77*	152	16,383.340	6.800	31.650	113.090	0.912	Cell membrane
AH19G31300.1	*AhCASP78*	75	8172.800	9.700	25.450	123.330	0.681	Cell membrane/Chloroplast/Peroxisome
AH19G36900.1	*AhCASP79*	195	21,762.850	8.930	42.000	90.050	0.251	Cell membrane
AH20G32430.1	*AhCASP80*	191	20,292.920	9.300	27.180	122.510	0.721	Cell membrane

Note. Length: length of amino acid (aa); pI: isoelectric point; GRAVY: grand average of hydropathicity.

**Table 2:** Non-synonymous (Ka) and synonymous (Ks) substitution rates of homologous *AhCASPs* gene pairs.

Gene 1	Gene 2	Ka	Ks	Ka/Ks	Divergence Time (Mya)
*AhCASP1*	*AhCASP39*	0.0028	0.0445	0.0628	2.62
*AhCASP2*	*AhCASP43*	0.0095	0.0076	1.2581	0.45
*AhCASP3*	*AhCASP42*	0.0074	0.0436	0.1695	2.56
*AhCASP1*	*AhCASP64*	0.1117	1.0843	0.1030	63.78
*AhCASP3*	*AhCASP69*	0.4946	2.5809	0.1917	151.82
*AhCASP17*	*AhCASP31*	0.2680	0.9484	0.2826	55.79
*AhCASP16*	*AhCASP54*	0.0794	0.1720	0.4616	10.12
*AhCASP17*	*AhCASP55*	0.0087	0.0445	0.1943	2.62
*AhCASP18*	*AhCASP56*	0.0167	0.0451	0.3699	2.65
*AhCASP13*	*AhCASP50*	0.0053	0.0337	0.1581	1.98
*AhCASP14*	*AhCASP51*	0.0326	0.0438	0.7452	2.58
*AhCASP12*	*AhCASP49*	0.1079	0.1875	0.5753	11.03
*AhCASP10*	*AhCASP48*	0.0023	0.0668	0.0338	3.93
*AhCASP17*	*AhCASP73*	0.2679	0.9053	0.2959	53.25
*AhCASP20*	*AhCASP59*	0.0066	0.0284	0.2305	1.67
*AhCASP21*	*AhCASP39*	0.1117	1.0843	0.1030	63.78
*AhCASP25*	*AhCASP40*	0.2872	1.1272	0.2548	66.31
*AhCASP21*	*AhCASP64*	0.0029	0.0269	0.1080	1.58
*AhCASP22*	*AhCASP65*	0.0186	0.0799	0.2327	4.70
*AhCASP23*	*AhCASP68*	0.0103	0.0468	0.2193	2.75
*AhCASP24*	*AhCASP67*	0.0277	0.0700	0.3961	4.12
*AhCASP25*	*AhCASP66*	0.0051	0.0394	0.1290	2.32
*AhCASP29*	*AhCASP71*	0.0110	0.0070	1.5832	0.41
*AhCASP30*	*AhCASP72*	0.0084	0.0075	1.1192	0.44
*AhCASP31*	*AhCASP55*	0.2629	0.9212	0.2854	54.19
*AhCASP32*	*AhCASP74*	0.0100	0.0189	0.5282	1.11
*AhCASP36*	*AhCASP77*	0.0058	0.0370	0.1574	2.18
*AhCASP37*	*AhCASP79*	0.0046	0.0487	0.0944	2.86
*AhCASP38*	*AhCASP80*	0.0191	0.0342	0.5605	2.01
*AhCASP40*	*AhCASP66*	0.2843	1.1055	0.2571	65.03
*AhCASP39*	*AhCASP64*	0.1151	1.0131	0.1136	59.59
*AhCASP55*	*AhCASP73*	0.2628	0.8794	0.2988	51.73

## Data Availability

Data is contained within the article.
